# Esthetic Rehabilitation of a Young Patient With Immediately Loaded Single-Piece Basal Implants Following a Trauma Involving the Anterior Maxilla: Case Report With Five-Year Follow-Up

**DOI:** 10.7759/cureus.42782

**Published:** 2023-08-01

**Authors:** Shakir Ahmed R, Athiban Inbarajan, Anusha KS, Kapil Raju, Maheshwaran KS

**Affiliations:** 1 Prosthodontics, Sri Ramachandra Dental College and Hospital, Sri Ramachandra Institute of Higher Education and Research, Chennai, IND

**Keywords:** single-piece implants, dental trauma, dental prosthesis, basal implants, dental implants

## Abstract

Traumatic injuries to the anterior maxilla usually lead to loss of teeth by means of avulsion or extraction due to fracture. Rehabilitation of such a clinical scenario is complex as it involves various anatomic and esthetic challenges. Single-piece basal implants have been widely used in the rehabilitation of resorbed ridges because they gain adequate anchorage from the basal cortical bone, allowing immediate temporization or loading. However, the use of basal implants in the anterior esthetic zone is not much documented. This case report with a five-year follow-up period describes the rehabilitation of lost teeth in the anterior esthetic zone of the anterior maxilla caused due to trauma following a train accident using single-piece axial basal implants with immediate temporization.

## Introduction

Management of the edentulous anterior maxilla following trauma possesses anatomic and esthetic challenges, including the condition of the buccal plate of the bone and the need for immediate temporary prosthesis for esthetic reasons. Basal implants consist of single-piece uni-body implants and are placed through the crestal approach, such as conventional endosseous implants gaining anchorage in the basal bone through active apical threads. Dr. Jean-Marc Julliet was the first one to use single-piece implants in 1972. Dr. Gerard Scortecci was the first to develop a single-piece implant system named "Diskimplants." Later, Dr. Stefan Idhe pioneered the development of basal osseointegrated implants (BOIs). Basal implants use the basal bone as an anchorage, which is less prone to resorption and infection as compared to the cortical bone utilized by conventional implants. In addition, conventional implants may require additional surgical procedure for augmentation, resulting in increased cost and duration of treatment [1]. Although basal implant-supported prosthesis has been well documented in the literature for ridge resorption and complete arch edentulous cases, studies on the use of basal implants in esthetic areas are lacking [2]. This case report describes a clinical scenario of the rehabilitation of missing teeth in the anterior maxilla following a train accident trauma using basal implants with immediate temporization and a five-year follow-up.

## Case presentation

**Figure 1:**
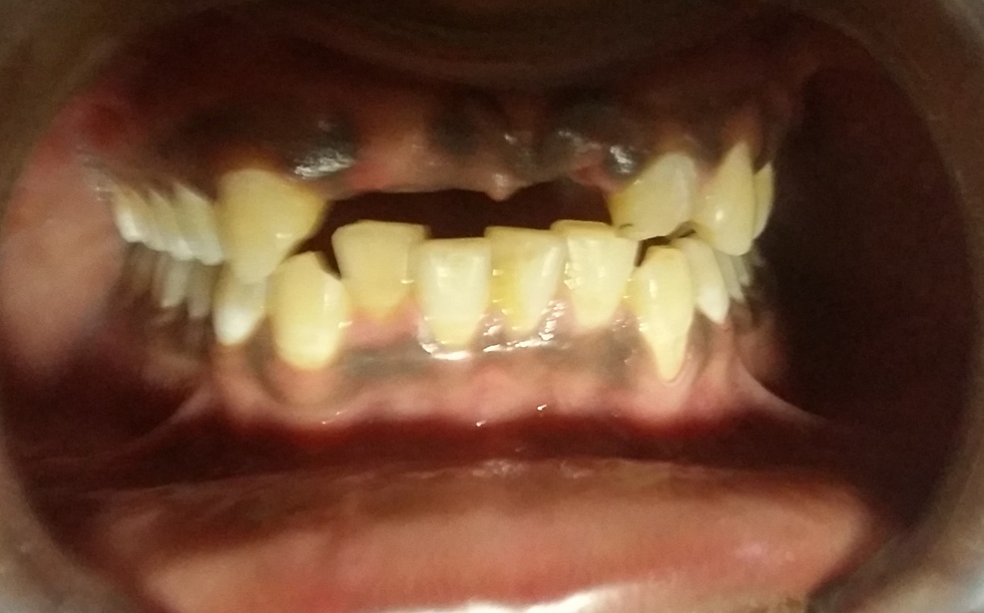
Pre-operative intra-oral photograph showing the edentulous maxillary anterior region in relation to tooth nos. 11, 12, and 21 and fractured tooth no. 22

A 21-year-old male reported a chief complaint of missing teeth in his upper front teeth region in the month of October 2016 and presented a history of trauma to his face due to a train accident that happened one month ago. Examination revealed missing teeth in relation to tooth nos. 11 and 12 and fractured tooth in relation to tooth no. 22 along with minimal loss of bone in the labial aspect of the crestal bone in relation to tooth nos. 11 and 12 (Figure 1).

**Figure 2:**
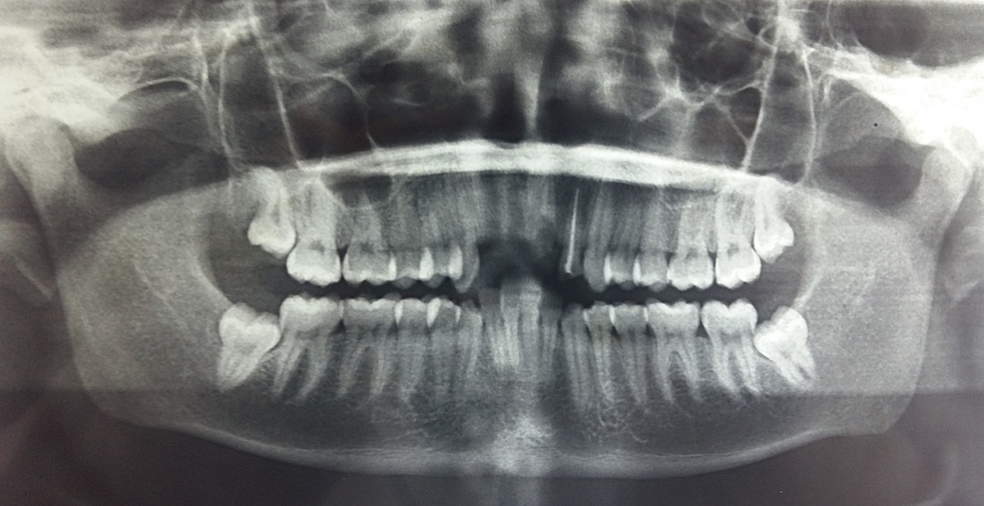
Pre-operative radiograph reveals radiolucency indicative of the recently extracted socket in relation to tooth nos. 11, 12, and 21 and radio-opaque filling material in relation to tooth no. 22 indicative of a root canal treatment OPG: orthopantomagram

Cone-beam computed tomography (CBCT) was not taken due to non-availability of the facility during the period of treatment planning and the patient's affordability. Panoramic radiograph revealed an extraction socket in relation to tooth nos. 11 and 12, root stump in tooth no. 21, and fractured tooth involving enamel, dentin, and approximating pulp in relation to tooth no. 22 (Figure 2).

**Figure 3:**
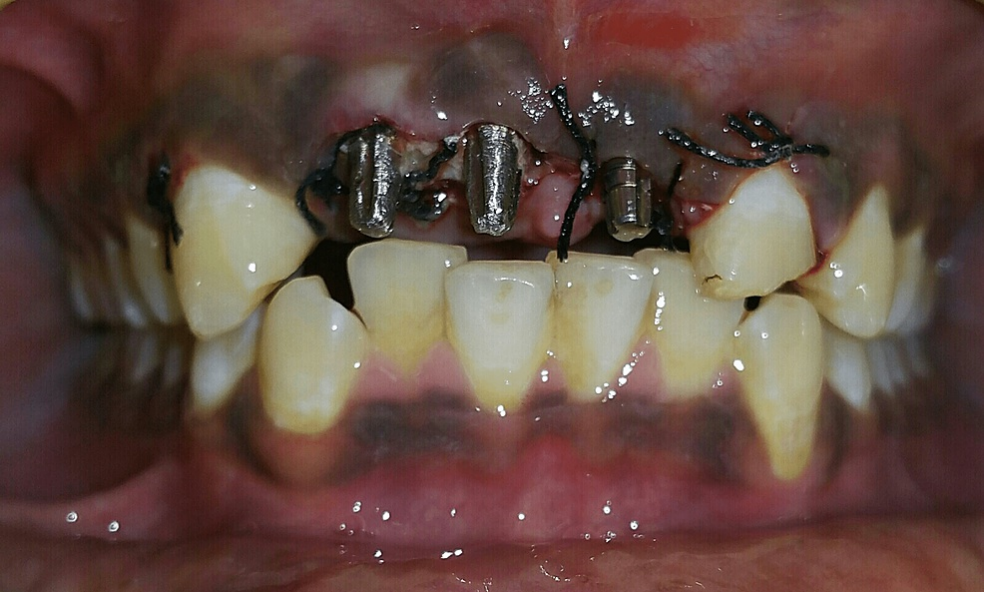
Immediate post-operative photograph showing single-piece abutments of implants placed in relation to the tooth no. 11, 12, and 21 region

Treatment options discussed included rehabilitation using conventional implants with bone augmentation, tooth-supported fixed dental prosthesis, and basal implant-supported fixed prosthesis. It was decided with the patient's consent to proceed with implant-supported fixed dental prosthesis using axial basal polished surface implants with cortical anchorage, considering that the clinically evident bone loss was evaluated by bone sounding in the labial aspect of the crestal bone and that the placement site had underwent recent extraction/avulsion and site in relation to tooth no. 21 requiring extraction of the root stump followed by immediate placement of the implant. The selection of single-piece basal implants was also made by taking the patient's affordability into account. A mock-up was done, but no template was used for implant placement. Root canal treatment was performed in relation to tooth no. 22, crestal incision was placed, and flaps were elevated in relation to tooth nos. 11, 12, and 21. On reflection of the flap, the loss of the labial plate of the bone in relation to the tooth no. 11 and 12 region to an extent of 2-3 mm was evident. Two basal cortical implants of GenXT implants (Regenics, India) of size 3.5 x 13 mm were placed in relation to tooth nos. 11 and 12; root stumps were extracted; immediate implant of a GenXT implant (Regenics, India) of size 3.5 x 13 mm was placed in tooth no. 21; flaps were approximated; and sutures were placed (Figure 3). 

**Figure 4:**
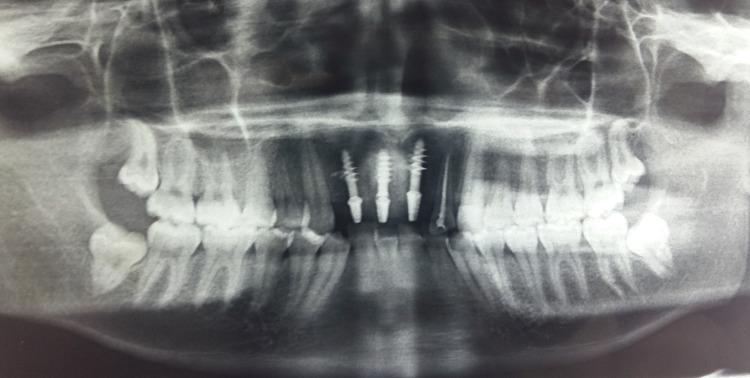
Post-operative radiograph revealed the presence of three radio-opaque structures in relation to tooth nos. 11, 12, and 21 indicative of dental implants

Post-operative radiograph revealed ideal positioning of Implants in relation to tooth nos. 11, 12, and 21 (Figure 4).

**Figure 5:**
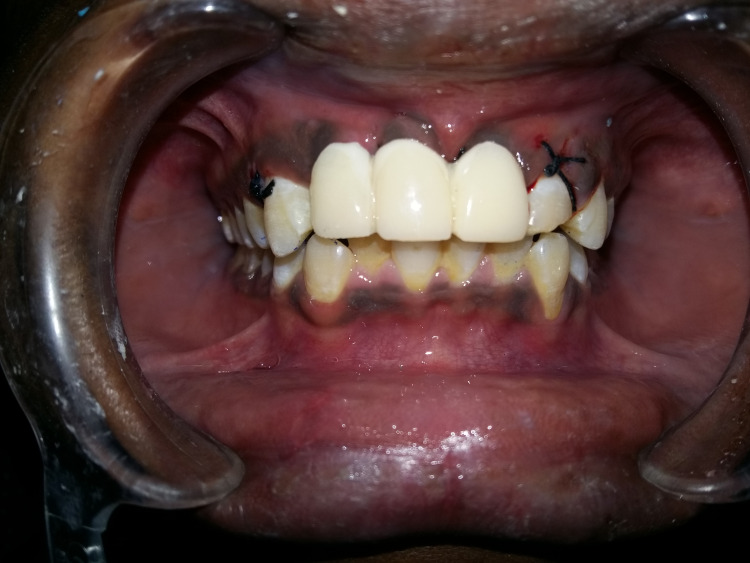
Intra-oral photograph showing immediate acrylic provisional restoration done in relation to the implants placed in tooth nos. 11, 12, and 21

Abutments were prepared, impression was made with alginate, and immediate temporization using CoolTemp bis-acryl composite (Coltene, Switzerland) was done and replaced with a long-term temporary acrylic fixed prosthesis the next day. Temporization was done using a three-point articulator, and guidance was adjusted and established clinically (Figure 5).

**Figure 6:**
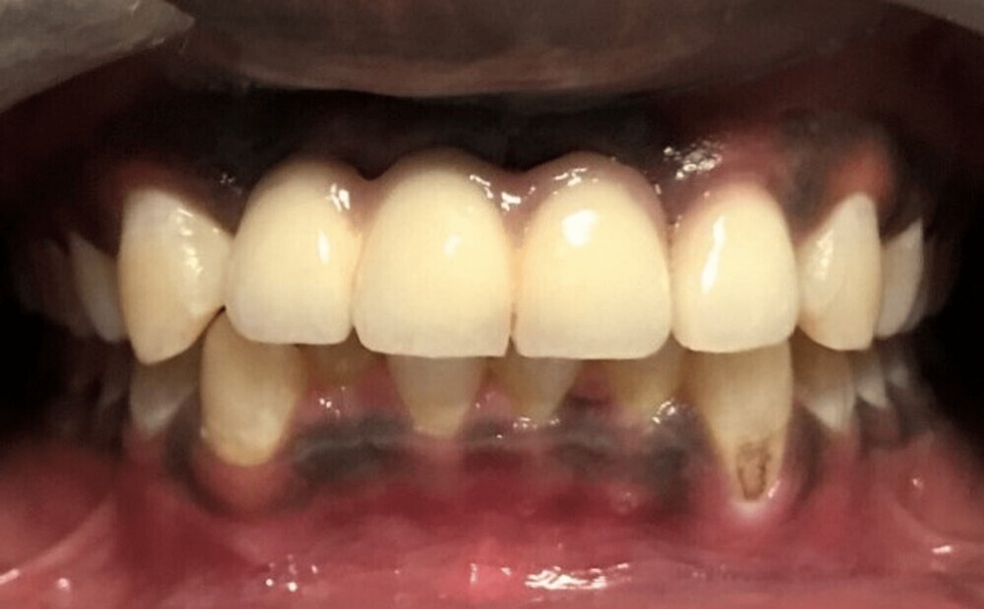
Intra-oral photograph showing three-unit metal ceramic prosthesis in relation to implants in the tooth nos. 11, 12, and 21 region and single metal-ceramic crown in relation to tooth no. 22

The patient was followed up after four months; implant abutments in relation to tooth nos. 11, 12, and 21 and the root canal treated tooth in relation to tooth no. 22 were prepared; and final impression was made using additional silicone putty and light body material. Three-unit metal-ceramic fixed dental prosthesis in relation to tooth nos. 11, 12, and 21 and single-unit crown in relation to tooth no. 22 were tried in, verified for fitting and esthetics, and were cemented using type I luting glass ionomer cement (Figure 6).

**Figure 7:**
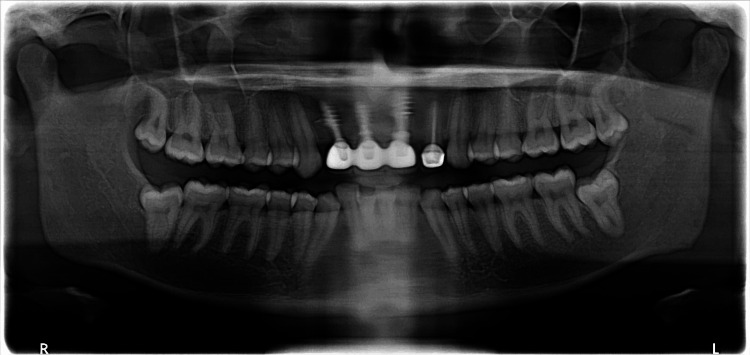
Five-year follow-up radiograph revealing adequate bones in relation to implants placed in the tooth nos. 11, 12, and 21 region

Post-insertion care instructions and follow-up schedule were given to the patient. The patient was recently followed up after five years, and on clinical evaluation, the prosthesis was found to be satisfactory in terms of esthetics, gingival margin position, and patient acceptance. The radiograph revealed adequate amount of bones around all the three implants with a very minimal amount of crestal bone loss (Figure 7).

## Discussion

The single-piece basal implants used in this case presented few advantages over conventional implants as they required no bone augmentation, thereby avoiding additional surgical procedure, reducing treatment cost and duration, and facilitating immediate loading using a temporary restoration. However, the use of conventional implant systems could have resulted in even more esthetically pleasing restorations with a better emergence profile and eliminated the need for gingival porcelain using components, such as a gingival former and/or angulated abutments as required. Basal cortical implants are available as single-piece implants. Thus, they eliminate microgaps between the abutment and implant [3]. They gain bone anchorage with the apical blade-like threads achieving greater primary stability than conventional implants. Immediate/early placement of single-piece basal implants following extraction or avulsion of teeth in the anterior esthetic zone provides a scope for immediate loading, thereby maintaining the patient’s esthetics using fixed temporary prostheses.

Adequately stabilized single-piece implants can be successfully loaded out of occlusion at the time of implant placement and definitively loaded in occlusion three months later without adversely affecting the function or esthetics [4]. Prithivraj et al. suggested that most surgeons preferred immediately loading single-piece implants [5]. Lazarov et al. reported a cumulative survival rate of 97.5% after four years for basal implants, and they also concluded that the immediate functional loading concept with cortically anchored implants for completely edentulous arches, segments, and single‑tooth replacement can be a viable concept even in cases where extractions of teeth were done simultaneously [6]. One-piece implants proved to be useful for tooth replacement in the posterior region and esthetic zone and also demonstrated long-term results of up to 10 years, proving that preserving the crestal bone level and biological width for periodontal stability can be realized [7]. Fadia et al. in their clinical study reported marked improvements in patients’ overall satisfaction and specific satisfaction with comfort, esthetics, mastication, and speech [8]. The design of basal implants to achieve cortical bone anchorage indicates their use in challenging clinical scenarios, including rehabilitation of atrophic ridges. Basal implants can play a vital role in the rehabilitation of patients, where compromised quality and/or quantity of bones is present [9].

The literature reports that the use of basal implants has been successful in peculiar clinical conditions, including single-tooth replacement in a patient with hyperdense lesions [10], full-mouth rehabilitation of a patient with cleidocranial dysplasia [11], rehabilitation of a patient who undergwent marginal mandibulectomy following oral squamous cell carcinoma [12], and rehabilitation of a patient with gunshot wounds in the mandible [13]. The case reported in this article also involves a clinical scenario of rehabilitating a patient with tooth loss following a trauma to the maxilla due to a train accident with axial basal implants, taking into consideration the patient's affordability, minimal loss of the labial aspect of the crestal bone, and need for immediate temporization. During the five-year follow-up evaluation, there was no bleeding on the probing or probing pocket depth or any other signs of peri-implantitis, and the patient was satisfied with both the esthetics and function of the restoration. 

The limitations of this case report include the unavailability of CBCT or intraoral periapical radiograph to compare the bone level, which was compared only by a clinical evaluation in this study.

## Conclusions

The maxillary anterior region is a complex zone. When the rehabilitation of edentulous teeth is planned, localized trauma and avulsion of the teeth resulting in a thin labial cortical bone add to the complexity. This case of rehabilitation using single-piece basal implants in the maxillary anterior region post trauma reported with a five-year follow-up indicates minimal or negligible bone loss around the implants, no incidence of peri-implantitis, and great patient acceptance and satisfaction. Case selection holds the key in choosing between basal and compressive implant systems, taking into account the advantages and disadvantages of both systems. Future long-term clinical trail studies comparing the outcomes of conventional and basal implants are needed.
